# Supplementary material for: Dual activities of an X-family DNA polymerase regulate CRISPR-induced insertional mutagenesis across species
Source: Nat Commun. 2024 Jul 26;15:6293. doi: 10.1038/s41467-024-50676-4 (PMC11282277; doi:10.1038/s41467-024-50676-4)
Supplement: Supplementary file 1 — Supplementary Information [file 41467_2024_50676_MOESM1_ESM.pdf]

Supplementary information

Dual activities of an X-family DNA polymerase regulate CRISPR-induced insertional mutagenesis across species

Trevor Weiss[†1, 2, 3, 4], Jitesh Kumar[†1, 2, 3, 4], Chuan Chen[5], Shengsong Guo[1,2], Oliver Schlegel[6], John Lutterman[6], Kun Ling[5], Feng Zhang[1, 2, 3, 4 *]

[†]Authors contributed equally

[*]Author for correspondence: zhangumn@umn.edu

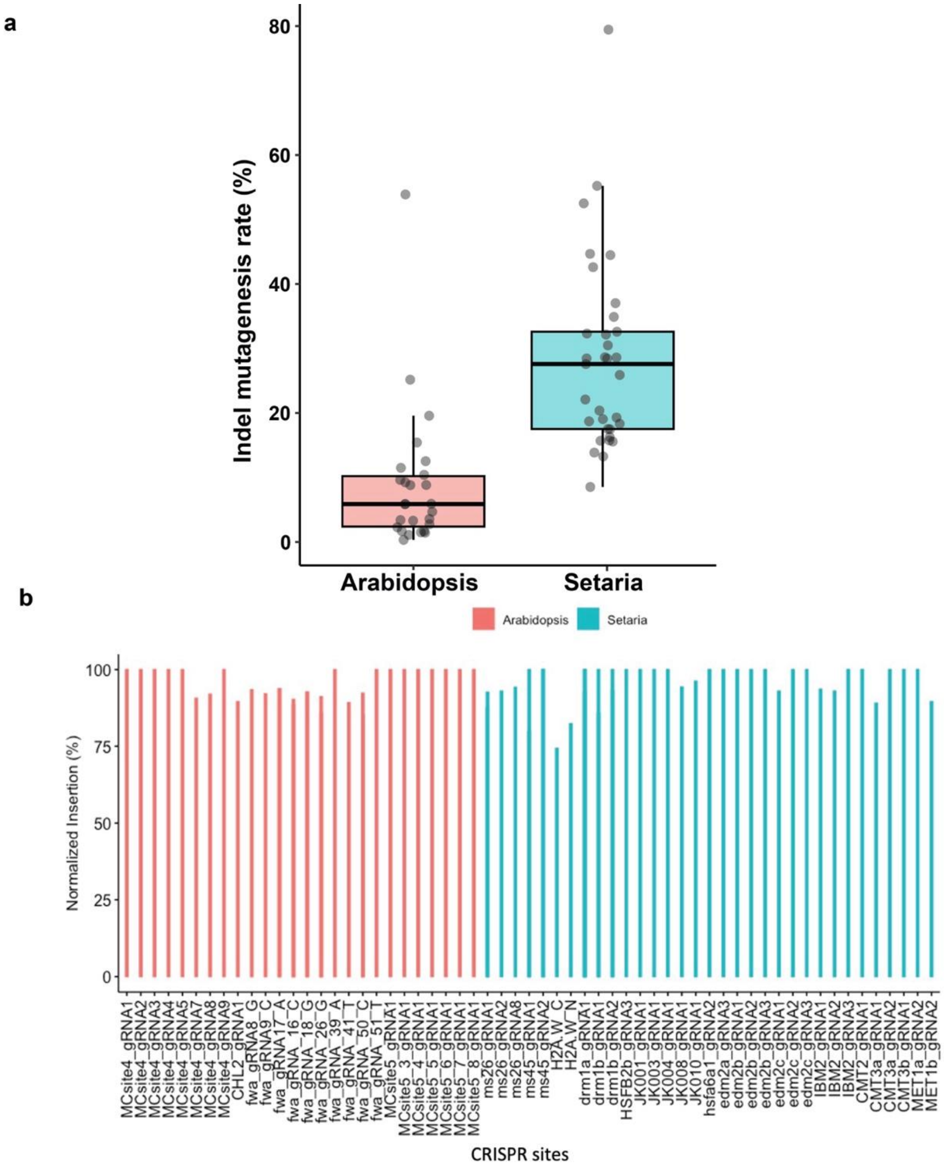

Supplementary Figure 1. Predominant 1-bp insertion patterns at the CRISPR sites in plants. **a.** The overall indel mutation rates across 59 sites in *Arabidopsis* (26 sites) and *Setaria* (33 sites). The indel rates (Y-axis) were determined by dividing the number of reads containing indel mutations by the total number of reads in each NGS sample. The horizontal bars within boxes represent medians. The top and bottom edges of the boxes represent the 75th and 25th percentiles, respectively. The upper and lower whiskers extend to data no more than 1.5x the interquartile range from the upper edge and lower edge of the box, respectively. **b.** Normalized 1-bp insertion rates (Y-axis) were plotted for individual CRISPR sites from *Arabidopsis* (26 sites; n=1 for each site) and *Setaria* (33 sites; n=1 for each site). The normalized 1-bp insertion rates were determined by dividing the number of reads containing 1-bp insertions by the total number of reads containing all types of insertional mutations. The source data are provided in the Source Data file.

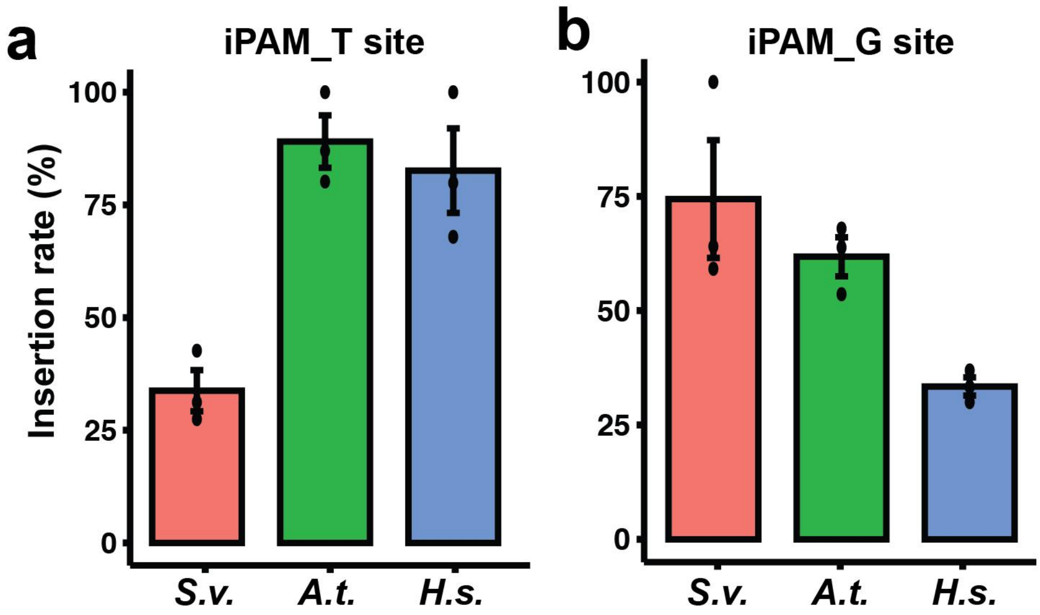

Supplementary Figure 2. Cross-species normalized insertion rates at the identical iPAM sites. The normalized insertion rates (Y-axis) were determined by dividing the number of reads containing 1-bp insertions by the number of reads with all types of indel mutations at iPAM_T site (**a**) and iPAM_G site (**b**). Values in the bar charts are means ± standard error of the mean (SEM) from 3 independent samples (n=3). The source data are provided in the Source Data file.

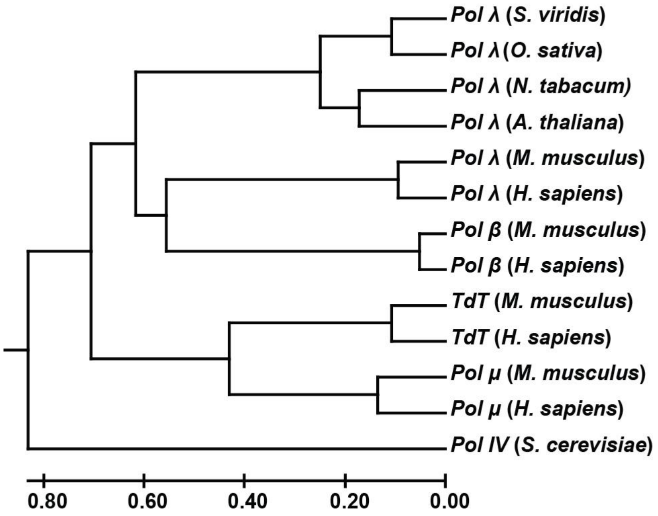

Supplementary Figure 3. Phylogeny of X-family DNA polymerase across species. Protein sequences of X-family DNA polymerases were obtained from human (*H. sapiens*), mouse (*M. Musculus*), *Arabidopsis* (*A. thaliana*), tobacco (*N. tabacum*), *Setaria* (*S. viridis*), rice (*O. sativa*), and budding yeast (*S. cerevisiae*). The neighbor-joining tree was generated using MEGA (version 11) with the default setting[1]. The DNA Pol IV from yeast was used as the outgroup.

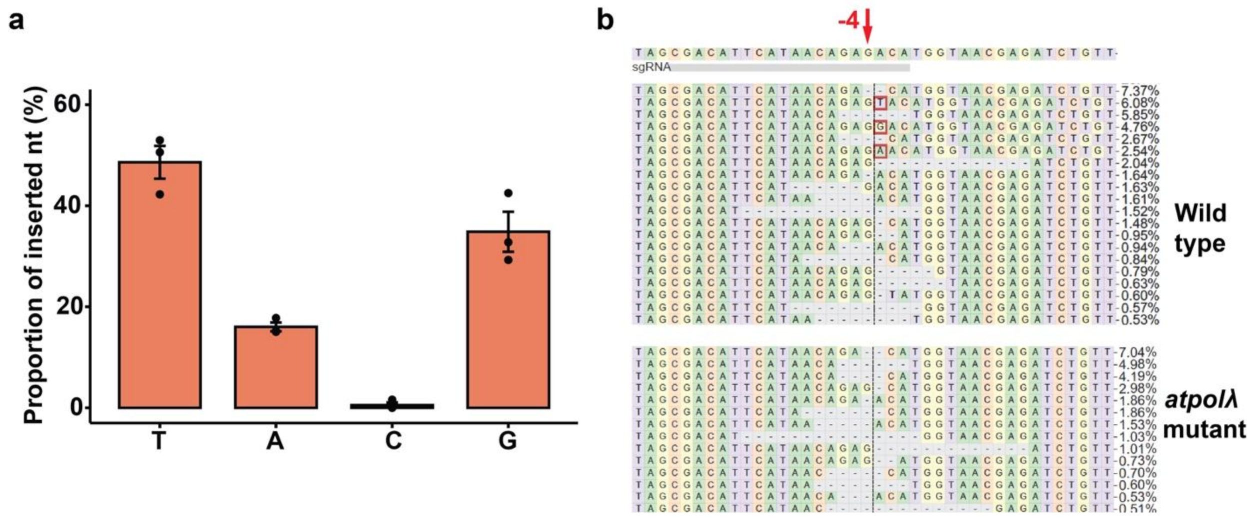

Supplementary Figure 4. DNA Pol λ is responsible for 1-bp insertions in *Arabidopsis*. **a.** 1-bp insertion rates at the CHLI2 site in the *Arabidopsis* wild-type and *atpolλ*-1 mutant plants. The fraction of each insertion type (Y-axis) was determined by dividing the number of reads containing individual 1-bp insertions by the number of reads with all types of 1-bp insertions. Values in the bar charts are means ± standard error of the mean (SEM) from 3 independent samples (n=3). **b.** The CRISPR-Cas9 mutation profile outputs at CHLI2 site from *Arabidopsis* wild-type and *atpolλ* mutant plants. The alignment outputs were generated by CRISPRESSO2 [2]. The 20 bp sgRNA targeted sequence highlighted by the gray bar. The -4th nucleotide of the target site was indicated by the red arrowhead. The 1-bp insertions were indicated by the red boxes while the deletions were indicated by the dash lines. The source data are provided in the Source Data file.

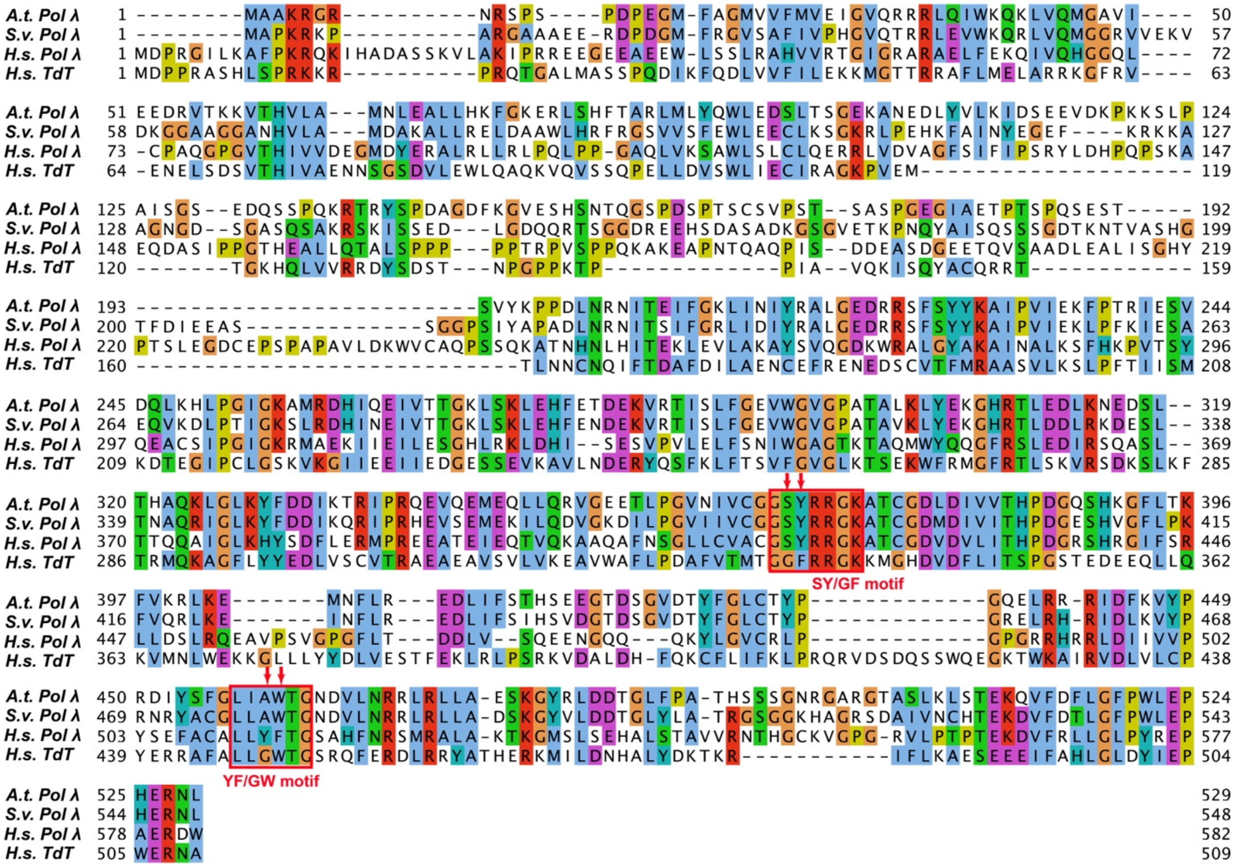

Supplementary Figure 5. Protein sequence alignment of DNA Pol λ and TdT across species. Protein sequences of DNA Pol λ and TdT from *Arabidopsis*, *Setaria* and human were aligned using the MUSCLE algorithm through MEGA (version 11) [3]. Two conserved motifs, SY/GF and YF/GW motifs, were highlighted with red boxes with the characteristic amino acids indicated by the red arrowheads.

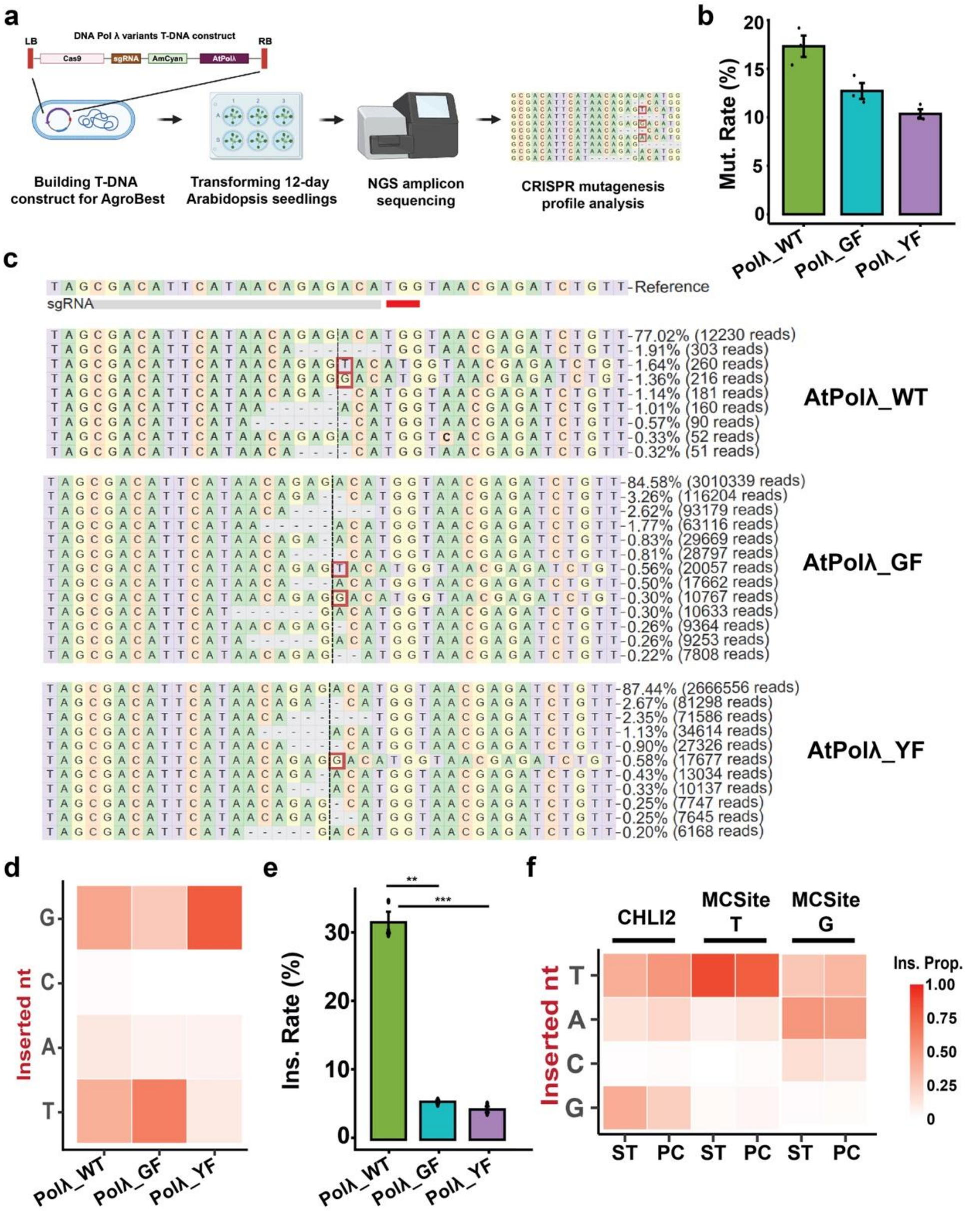

Supplementary Figure 6. Functional analyses of AtDNA Pol λ variants. **a.** Flowchart of the AGROBEST-based CRISPR mutagenesis procedure. **b.** Comparisons of the overall mutation rates between the wild type AtDNA Pol λ and two variants, Pol λ$^{S366G/Y367F}$ and Pol λ$^{A459Y/W460F}$ at the CHLI2 site. The mutation rates (Y-axis) were determined by dividing the number of reads containing indel mutations by the total number of NGS reads. Values in the bar charts are means ± standard error of the mean (SEM) from 3 independent samples. **c.** The examples of CRISPR-Cas9 mutation profiles at CHLI2 site from the homozygous *atpolλ* mutant plants transformed with the wild type AtDNA Pol λ and two variants, Pol λ$^{S366G/Y367F}$ and Pol λ$^{A459Y/W460F}$. The alignment outputs were generated by CRISPRESSO2 [2]. The 20 bp sgRNA targeted sequence highlighted by the gray bar. The PAM sequence was underlined by the red bar. The 1-bp insertions were indicated by the red boxes while the deletions were indicated by the dash lines. **d.** Heatmap analyses of the proportions for each type of inserted nucleotide at the -4th position of

the CHLI2 site for the homozygous *atpolλ* mutant plants transformed with the wild type AtDNA Pol λ and two variants. Three replications were conducted for each group. **e.** Comparisons of the normalized 1-bp insertion rates between the wild type AtDNA Pol λ and two variants at the CHLI2 site. The 1-bp insertion rates (Y-axis) were determined by dividing the number of reads containing 1-bp insertions by the number of reads with all types of indel mutations. Values in the bar charts are means ± standard error of the mean (SEM) from 3 independent samples (n=3). Statistical significance was represented by *P*-values (*, $P \leq 0.05$; **, $P \leq 0.01$; ***, $P \leq 0.001$). **f.** Heatmap analyses of the proportions of each inserted nucleotide type at the -4th position of three CRISPR sites between the stable transgenic plants (ST) and transfected protoplast cells (PC). The source data are provided in the Source Data file. **a** created with BioRender.com released under a Creative Commons Attribution-NonCommercial-NoDerivs 4.0 International license.

Supplementary References

1. Tamura, K., Stecher, G. & Kumar, S. MEGA11: Molecular Evolutionary Genetics Analysis Version 11. *Mol. Biol. Evol.* **38**, 3022–3027 (2021).
2. Clement, K. *et al.* CRISPResso2 provides accurate and rapid genome editing sequence analysis. *Nat. Biotechnol.* **37**, 224–226 (2019).
3. Edgar, R. C. MUSCLE: multiple sequence alignment with high accuracy and high throughput. *Nucleic Acids Res.* **32**, 1792–1797 (2004).
