# Supplementary material for: Dual activities of an X-family DNA polymerase regulate CRISPR-induced insertional mutagenesis across species
Source: Nat Commun. 2024 Jul 26;15:6293. doi: 10.1038/s41467-024-50676-4 (PMC11282277; doi:10.1038/s41467-024-50676-4)
Supplement: Supplementary file 3 — Description of Additional Supplementary Files [file 41467_2024_50676_MOESM3_ESM.pdf]

Description of Additional Supplementary Files

File Name: Supplementary Data 1

Description: Information of each CRIPSR targeted site was listed from *Arabidopsis thaliana*, *Setaria viridis*, and Human cells.

File Name: Supplementary Data 2

Description: Protein sequences of X-family DNA Polymerases from 8 species, including yeast, *Arabidopsis thaliana*, *Setaria viridis*, *Nicotiana tabacum*, rice, human and mouse.

File Name: Supplementary Data 3

Description: Summary of oligonucleotide sequences for plasmid construction and PCR primers.

File Name: Supplementary Data 4

Description: Summary of overall mutation rates for each CRISPR targeted site tested in Figures 2d, 3a-b, 4a, 4d-e, 5a-b, Supplementary Figures 1, 6b and 6f. The overall mutation rates were determined by dividing the number of reads containing indel mutations by the total number of sequencing reads.
