# Supplementary material for: Dual activities of an X-family DNA polymerase regulate CRISPR-induced insertional mutagenesis across species
Source: Nat Commun. 2024 Jul 26;15:6293. doi: 10.1038/s41467-024-50676-4 (PMC11282277; doi:10.1038/s41467-024-50676-4)
Supplement: Supplementary file 8 — Reporting Summary [file 41467_2024_50676_MOESM8_ESM.pdf]

nature portfolio

Corresponding author(s): Feng Zhang

Last updated by author(s): 2024/05/07

Reporting Summary

Nature Portfolio wishes to improve the reproducibility of the work that we publish. This form provides structure for consistency and transparency in reporting. For further information on Nature Portfolio policies, see our Editorial Policies and the Editorial Policy Checklist.

Statistics

For all statistical analyses, confirm that the following items are present in the figure legend, table legend, main text, or Methods section.

n/a	Confirmed	
☐	☒	The exact sample size (n) for each experimental group/condition, given as a discrete number and unit of measurement
☐	☒	A statement on whether measurements were taken from distinct samples or whether the same sample was measured repeatedly
☐	☒	The statistical test(s) used AND whether they are one- or two-sided<br>*Only common tests should be described solely by name; describe more complex techniques in the Methods section.*
☒	☐	A description of all covariates tested
☐	☒	A description of any assumptions or corrections, such as tests of normality and adjustment for multiple comparisons
☐	☒	A full description of the statistical parameters including central tendency (e.g. means) or other basic estimates (e.g. regression coefficient) AND variation (e.g. standard deviation) or associated estimates of uncertainty (e.g. confidence intervals)
☐	☒	For null hypothesis testing, the test statistic (e.g. F, t, r) with confidence intervals, effect sizes, degrees of freedom and P value noted<br>*Give P values as exact values whenever suitable.*
☒	☐	For Bayesian analysis, information on the choice of priors and Markov chain Monte Carlo settings
☒	☐	For hierarchical and complex designs, identification of the appropriate level for tests and full reporting of outcomes
☐	☒	Estimates of effect sizes (e.g. Cohen's d, Pearson's r), indicating how they were calculated

Our web collection on statistics for biologists contains articles on many of the points above.

Software and code

Policy information about availability of computer code

Data collection	N.A.
Data analysis	The softwares used in this study include: CRISPResso2 (version 2.1.1; doi: 10.1038/s41587-019-0032-3), R studio (Version 2023.09.1) and MEGA (version 11).

For manuscripts utilizing custom algorithms or software that are central to the research but not yet described in published literature, software must be made available to editors and reviewers. We strongly encourage code deposition in a community repository (e.g. GitHub). See the Nature Portfolio guidelines for submitting code & software for further information.

Data

Policy information about availability of data

All manuscripts must include a data availability statement. This statement should provide the following information, where applicable:

- Accession codes, unique identifiers, or web links for publicly available datasets
- A description of any restrictions on data availability
- For clinical datasets or third party data, please ensure that the statement adheres to our policy

The plasmids used in the Golden Gate assembly are available from Addgene with the ID number listed in the Methods. The new plasmids made in this manuscript will be deposited to Addgene. The Next-Generation sequencing data sets are available at NCBI Sequence Read Archive under Bioproject PRJNA1062985. The CRISPRESSO output files for each sample can be downloaded through the link: https://github.com/ZhangLab-UMN/Weiss_Kumar_1bp_manuscript.git.

Research involving human participants, their data, or biological material

Policy information about studies with human participants or human data. See also policy information about sex, gender (identity/presentation), and sexual orientation and race, ethnicity and racism.

Reporting on sex and gender	N.A.
Reporting on race, ethnicity, or other socially relevant groupings	N.A.
Population characteristics	N.A.
Recruitment	N.A.
Ethics oversight	N.A.

Note that full information on the approval of the study protocol must also be provided in the manuscript.

Field-specific reporting

Please select the one below that is the best fit for your research. If you are not sure, read the appropriate sections before making your selection.

☒ Life sciences ☐ Behavioural & social sciences ☐ Ecological, evolutionary & environmental sciences

For a reference copy of the document with all sections, see nature.com/documents/nr-reporting-summary-flat.pdf

Life sciences study design

All studies must disclose on these points even when the disclosure is negative.

Sample size	Sample sizes were determined based on requirements for sufficient statistical power, as established by relevant published reports in the field (DOI: 10.1038/s41467-024-49473-w; DOI: 10.1038/s41467-022-29130-w; DOI: 10.1093/plcell/koad139). The sample size for each experiment was fully described in either the figure legends or the Methods section.
Data exclusions	No data was excluded from this study.
Replication	To verify the reproducibility of CRISPR-Cas9 mediated mutagenesis in each experiment , three biological replications were performed for each experiment. All attempts at replication were successful.
Randomization	The plant and human cells were collected randomly from the subject materials. All samples were randomly allocated across all replicates.
Blinding	The investigators were blinded to group allocation during data collection and/or analysis for all experiments.

Reporting for specific materials, systems and methods

We require information from authors about some types of materials, experimental systems and methods used in many studies. Here, indicate whether each material, system or method listed is relevant to your study. If you are not sure if a list item applies to your research, read the appropriate section before selecting a response.

Materials & experimental systems

n/a	Involved in the study	
☒	☐	Antibodies
☐	☒	Eukaryotic cell lines
☒	☐	Palaeontology and archaeology
☒	☐	Animals and other organisms
☒	☐	Clinical data
☒	☐	Dual use research of concern
☐	☒	Plants

Methods

n/a	Involved in the study	
☒	☐	ChIP-seq
☒	☐	Flow cytometry
☒	☐	MRI-based neuroimaging

Eukaryotic cell lines

Policy information about cell lines and Sex and Gender in Research

Cell line source(s)	HEK293 (Cat# CRL-1573) and 293T (Cat# CRL-3216) cell lines were purchased from ATCC.
Authentication	We have not authenticated these cell lines since we purchased them from ATCC.
Mycoplasma contamination	Both cell lines are negative for mycoplasma contamination. Cells are subjected to DAPI staining and examined under Confocal microscope every 1-3 months. No DAPI signal was found outside of the nucleus.
Commonly misidentified lines (See ICLAC register)	It was reported once in 1981 that HEK293 cells were contaminated by HeLa cells. HEK293 and HeLa are two different cell lines carrying separate genetic identities, which is recognized by ATCC. Our two 293 cell lines were directly purchased from ATCC.

Dual use research of concern

Policy information about dual use research of concern

Hazards

Could the accidental, deliberate or reckless misuse of agents or technologies generated in the work, or the application of information presented in the manuscript, pose a threat to:

No	Yes	
☒	☐	Public health
☒	☐	National security
☒	☐	Crops and/or livestock
☒	☐	Ecosystems
☒	☐	Any other significant area

Experiments of concern

Does the work involve any of these experiments of concern:

No	Yes	
☒	☐	Demonstrate how to render a vaccine ineffective
☒	☐	Confer resistance to therapeutically useful antibiotics or antiviral agents
☒	☐	Enhance the virulence of a pathogen or render a nonpathogen virulent
☒	☐	Increase transmissibility of a pathogen
☒	☐	Alter the host range of a pathogen
☒	☐	Enable evasion of diagnostic/detection modalities
☒	☐	Enable the weaponization of a biological agent or toxin
☒	☐	Any other potentially harmful combination of experiments and agents

Plants

Seed stocks	Homozygous Arabidopsis DNA Pol λ knock-out line (atPolλ-1) was obtained from Arabidopsis Biological Resource Center (ABRC) with the stock number SALK_075391C.
Novel plant genotypes	The Arabidopsis transgenic lines were generated using the T-DNA-mediated floral dip transformation method. Transgenic T1 seeds were sown on soil and treated with BASTA selection to select transgenic plants. Three independent transgenic plants were used in each experiment.
Authentication	The homozygous Arabidopsis DNA Pol λ knock-out line (atPolλ-1) were confirmed using PCR primers provided by the Arabidopsis Biological Resource Center (ABRC). The mutation phenotype of this homozygous line can be restored by expressing the cDNA of the AtDNA Polλ gene using the transgenic approach. All transgenic plants were confirmed by the BASTA selection and the PCR assay using the primers of the BASTA gene.
